# Taro (*Colocasia esculenta* (L.) Schott) Corms and Cormels Cultivated in Brazil as Plant-Based Food Ingredients: Nutritional, Microstructural, and Pasting Properties

**DOI:** 10.1007/s11130-026-01544-7

**Published:** 2026-08-18

**Authors:** Natali Alcântara Brandão, Bruna Lago Tagliapietra, Rebeca Salvador-Reyes, Gustavo Costa do Nascimento, Juliana Azevedo Lima Pallone, Elisabeth Harumi Nabeshima, Maria Teresa Pedrosa Silva Clerici

**Affiliations:** 1 Department of Food Science and Nutrition, Faculty of Food Engineering, Universidade Estadual de Campinas, Campinas, SP 13083-862 Brazil; 2 Department of Food and Nutrition, Federal University of Santa Maria, Palmeira das Missões Campus, Palmeira das Missões, RS 98300-000 Brazil; 3 Facultad de Ingeniería, Universidad Tecnológica del Perú, Lima, 150101 Peru; 4 Institute of Food Technology – Center for Cereal and Chocolate Technology (Cereal Chocotec), Campinas, SP 13070178 Brazil

**Keywords:** *Colocasia esculenta*, Food security, Underutilized crops, Sustainable food systems, Climate-resilient crops, Starch functionality

## Abstract

Taro (*Colocasia esculenta* (L.) Schott) is a carbohydrate-rich tuber crop cultivated in Brazil, with potential for the development of plant-based food ingredients. However, taro corms are less commercially valued than cormels, despite their possible nutritional and technological relevance. This study compared the physicochemical, nutritional, microstructural, and pasting properties of taro corms (C), cormels (CL), and their isolated starches (CS and CLS). Cormels showed lighter and more homogeneous pulp, whereas corms had greater size and shape regularity. Nutritionally, corms contained higher total dietary fiber and calcium contents, while cormels showed higher protein, available carbohydrate, and potassium contents. SEM analysis revealed small starch granules with sub-spherical to polyhedral morphology, with CS showing larger average diameter, perimeter, and area than CLS. Regarding pasting properties, C, CL, CS, and CLS showed pasting temperatures close to 80 °C. CL exhibited higher peak, final, and setback viscosities, as well as greater gel hardness, suggesting suitability for products requiring structure and consistency. In contrast, C showed lower breakdown and setback, indicating greater thermal stability and lower retrogradation tendency. Isolated starches showed lower viscosities than the corresponding whole fractions, which may be associated with the removal of non-starch components. Overall, taro corms and cormels showed complementary nutritional and technological properties, supporting their potential use as differentiated plant-based ingredients for food applications and the valorization of underutilized taro fractions.

**Supplementary Information:**

The online version contains supplementary material available at https://doi.org/10.1007/s11130-026-01544-7.

## Introduction

 Taro (*Colocasia esculenta* (L.) Schott) is a tropical and subtropical root crop belonging to the Araceae family, widely recognized for its adaptability to low-input agricultural systems and its high carbohydrate content, mainly in the form of starch [1]. In Brazil, its nomenclature varies according to region and local use. *Colocasia esculenta* is commonly referred to as “inhame” in the South, Southeast, and Central-West regions, whereas the term “cará” is often associated with species of the genus *Dioscorea*, although botanical standardization has established *Colocasia esculenta* as taro and *Dioscorea* spp. as yam, these terms are still used inconsistently by farmers, consumers, and markets [2]. Therefore, the present study adopts the scientific nomenclature while acknowledging the traditional names used in Brazil.

The mature taro plant develops an underground stem composed of a central corm, also known as the mother rhizome or “head”, from which smaller cormels, also called daughter rhizomes or “fingers”, originate and contribute to plant propagation, as illustrated in Supplementary Material (SM) Fig. S1 [2–4]. However, these structures differ in size, morphology, postharvest stability, and commercial value. Corms are larger and may weigh between 100 and 1,000 g, whereas cormels are smaller, usually ranging from 30 to 250 g, with oval to elongated shapes and greater consumer preference due to their smaller size and ease of preparation [2, 3, 5].

Despite its relevance as a food crop, taro is highly perishable. Postharvest losses of corms and cormels have been estimated at around 30%, and their shelf life under dry, cool, and well-ventilated conditions is generally limited to 2–4 weeks [6, 7]. Corms are particularly susceptible to deterioration, with greater mass loss, firmness reduction, and early sprouting compared with cormels [7, 8]. In addition, the higher commercial preference for cormels contributes to the underutilization of corms, which often remain less visible within the taro production chain [4]. This scenario highlights the need to better characterize both fractions and identify processing strategies capable of increasing their storage stability, technological value, and food applications.

 Taro is of particular interest because of its high starch content, small starch granules, and presence of mucilage and dietary fiber. These components may influence water retention, viscosity, texture, and starch accessibility during digestion, supporting potential applications as natural thickening or structuring agents in food systems [9]. However, comparative information on the nutritional composition, microstructure, and pasting behavior of taro corms and cormels cultivated in Brazil remains limited. Therefore, this study aimed to compare the physicochemical, nutritional, microstructural, and pasting properties of taro corms and cormels cultivated in Brazil, including their isolated starches, to identify differentiated food applications and support the valorization of both fractions as plant-based ingredients.

## Materials and Methods

Taro (*Colocasia esculenta* (L.) Schott) was obtained from a local producer in Bom Repouso, Minas Gerais, Brazil. Plants were harvested nine months after planting and manually separated into corms (C) and cormels (CL). Part of each fraction was processed for starch extraction, yielding corm starch (CS) and cormel starch (CLS).

Fresh C and CL samples were used for physicochemical characterization, while oven-dried samples were used for nutritional composition analyses. Freeze-dried samples and isolated starches were evaluated by scanning electron microscopy (SEM). Pasting properties were determined using a Rapid Visco Analyzer (RVA) in dried flours and isolated starches. The isolated starches, CS and CLS, were obtained specifically for microstructural and pasting property analyses.

All analyses were performed at least in triplicate, and results were expressed as mean ± standard deviation. Comparisons between C and CL, and between CS and CLS when applicable, were performed using Student’s *t*-test at *p* < 0.05. Detailed information on sample preparation, analytical procedures, equipment, operating conditions, and statistical analysis is provided in Supplementary Material (Detailed Material and Methodology).

## Results and Discussion

### Color

**Fig. 1:**
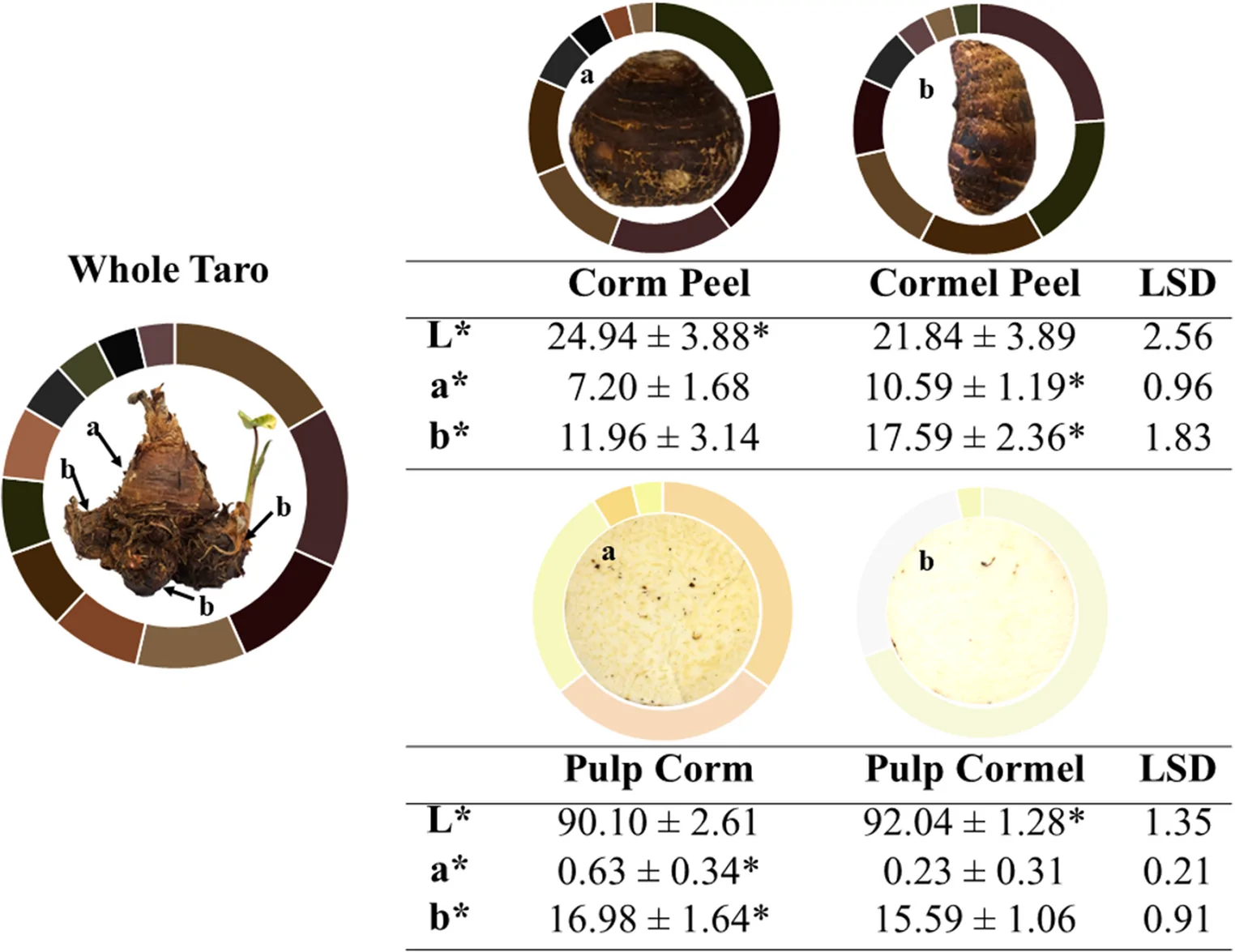
Color characteristics of fresh taro corms and cormels, including the whole plant, peel and pulp portions, CIE L*a*b* parameters, and representative RGB color distribution. Corms and cormels are identified as a and b, respectively. LSD: least significant difference; * indicates significant difference between corms and cormels for the same portion at *p* < 0.05. L* represents lightness, whereas a* and b* represent chromaticity from green to red and from blue to yellow, respectively

Figure 1 shows the visual appearance of fresh taro, including the whole plant and the peel and pulp portions of C and CL, together with their CIE L*a*b* color parameters and representative RGB color distribution. The peel of both fractions exhibited a predominantly brown color, ranging from light to dark brown. Corm peel showed significantly higher lightness (L* = 24.94 ± 3.88; *p* < 0.05) than cormel peel (L* = 21.84 ± 3.89). In contrast, cormel peel presented significantly higher a* (10.59 ± 1.19) and b* (17.59 ± 2.36) values than corm peel (a* = 7.20 ± 1.68; b* = 11.96 ± 3.14), indicating a greater contribution of reddish and yellowish tones. These differences in peel color may be associated with environmental factors, physiological development, or adaptive responses and may also influence external appearance and commercial acceptance [10].

In the pulp, cormels exhibited significantly higher lightness (L* = 92.04 ± 1.28; *p* < 0.05) than corms (L* = 90.10 ± 2.61). Conversely, corm pulp presented higher a* (0.63 ± 0.34) and b* (16.98 ± 1.64) values than cormel pulp (a* = 0.23 ± 0.31; b* = 15.59 ± 1.06), with significant differences between the fractions (*p* < 0.05). Overall, corms exhibited a darker cream-yellow pulp, whereas cormels displayed a lighter and more homogeneous pulp. The differences in peel and pulp color between corms and cormels may be associated with differences in the developmental stage of these storage organs, since cormels are lateral structures with distinct growth dynamics, whereas the corm represents the primary storage organ of the plant [11]. Developmental changes in taro storage organs are accompanied by significant physiological and biochemical modifications, including alterations in reserve accumulation and metabolism [12]. However, further studies evaluating pigment composition and other biochemical constituents are required to elucidate the mechanisms underlying the observed color differences.

### Physicochemical Properties

The physicochemical characteristics of corms and cormels of taro are shown in Table 1. C showed significantly (*p* < 0.05) greater average width (9.50 ± 1.08 cm) than CL (6.68 ± 1.33 cm), whereas both fractions presented similar average lengths. This indicates that the main morphological difference between C and CL was associated with transverse development rather than longitudinal growth. The distribution data also showed that corms were concentrated in larger width ranges 9.00–10.50 cm (52.63%), while cormels were more frequent in smaller size ranges 6.00–7.50 cm (63.16%), confirming their distinct morphology and commercial classification.

**Table 1:** Physicochemical characterization of taro corms (C) and cormels (CL)^#^

Properties	Samples		
	C	CL	LSD
Average width (cm)	9.50 ± 1.08*	6.68 ± 1.33	0.81
* Width distribution (cm)*	*Relative frequency (%)*		
[4.49–6.00>	0.00	21.05	
[6.00–7.50>	5.26	63.16	
[7.50–9.00>	26.32	5.26	
[9.00–10.50>	52.63	10.53	
[10.50–11.67>	15.79	0.00	
Average length (cm)	9.51 ± 1.28	9.49 ± 1.54	0.95
* Length distribution (cm)*	*Relative frequency (%)*		
[6.54–7.70>	5.26	15.79	
[7.70–8.90>	42.11	10.53	
[8.90–10.10>	10.53	31.58	
[10.10–11.30>	36.84	31.58	
[11.30–12.55>	5.26	10.53	
Average regularity^c^	0.88 ± 0.07*	0.62 ± 0.13	0.07
* Regularity distribution*	*Relative frequency (%)*		
[0.37–0.50>	0.00	15.79	
[0.50–0.63>	0.00	42.11	
[0.63–0.76>	5.26	26.32	
[0.76–0.89>	52.63	5.26	
[0.89–1.00>	42.11	10.53	
Weight (g)	355.62 ± 101.66*	133.53 ± 28.74	49.16
Specific gravity (g/mL)	0.97 ± 0.04	1.00 ± 0.05*	0.03
Moisture (%)	74.46 ± 5.96	75.11 ± 4.31	3.42
Dry Matter (%)	25.54 ± 5.96	24.89 ± 4.31	3.42
Soluble solids (°Brix)	5.74 ± 0.77	5.44 ± 0.91	0.56
pH	6.33 ± 0.16	6.35 ± 0.09	0.08
Titratable acidity (%)	2.42 ± 0.19	2.28 ± 0.35	0.19

^#^Results are presented as mean ± standard deviation. Distribution data are presented as relative frequency (%). In the distribution intervals, “[” indicates a closed limit and “>” indicates an open limit. The regularity parameter is dimensionless, ranging from 0 to 1, where values closer to 0 indicate a more heterogeneous shape and values closer to 1 indicate a more homogeneous or regular shape. *LSD* least significant difference; * indicates significant difference between C and CL at *p* < 0.05.

Corms also showed significantly higher regularity than cormels (*p* < 0.05), with an average value of 0.88 ± 0.07 compared with 0.62 ± 0.13 for CL. This indicates that corms had a more rounded and homogeneous morphology, whereas cormels were more elongated and less standardized. From a processing perspective, higher regularity may favor mechanical handling, cutting, cleaning, and equipment design. However, the greater weight and variability of corms may also require specific adjustments depending on the intended industrial application. Agronomic factors such as planting depth and spacing have been shown to influence corm length and yield, reinforcing that morphology may vary according to genotype and cultivation conditions [13].

Corms were significantly (*p* < 0.05) heavier than cormels, with average weights of 355.62 ± 101.66 g and 133.53 ± 28.74 g, respectively. The relationship between size, weight, and industrial suitability may vary according to the target product. Larger corms may be suitable for flour production, starch extraction, or institutional food processing, whereas medium-sized cormels are more commonly preferred for fresh consumption and small-scale culinary uses. Previous studies have shown that differences in taro size and weight may affect flour quality, including starch content and gelatinization-related properties [14]. Therefore, classification by size and morphology may be useful for improving raw material standardization, reducing postharvest losses, and defining differentiated processing routes for C and CL.

Regarding the physicochemical parameters, C and CL did not differ significantly in moisture, dry matter, soluble solids, pH, or titratable acidity. Moisture values were close to 75% in both fractions, indicating high water content and, consequently, limited postharvest stability. Paula [4] reported moisture contents of 84.18% for corms and 78.48% for cormels, suggesting that moisture may vary according to cultivar, maturity stage, cultivation conditions, and postharvest handling. In the present study, the similar moisture and dry matter contents of C and CL suggest comparable processing yields on a fresh-weight basis. Only specific gravity differed significantly between fractions, with CL showing a slightly higher value than C, which may reflect differences in tissue compactness or internal structure.

### Nutritional Composition

The proximate composition and mineral content of C and CL of taro are shown in Table 2. Significant differences between C and CL were observed for ash, protein, total dietary fiber, available carbohydrates, reducing sugars, calcium, magnesium, manganese, potassium, and zinc, indicating that both fractions have distinct and complementary nutritional profiles.

**Table 2:** Proximate composition and mineral content of taro corms (C) and cormels (CL)

Properties	Samples		
	C	CL	LSD
Centesimal composition (g/100 g dry base)			
Ash	6.63 ± 0.09	7.14 ± 0.13*	0.19
Lipids	0.99 ± 0.04	0.92 ± 0.06	0.08
Protein	5.83 ± 0.21	7.37 ± 0.11*	0.63
Total Dietary Fiber	23.27 ± 1.04*	17.36 ± 0.50	2.75
Digestible carbohydrates^c^	63.29 ± 1.21	67.21 ± 0.43*	2.30
Carbohydrates into glucose (g/ 100 g dry base)			
Totals	49.03 ± 2.54	46.76 ± 2.91	7.95
Reducers	6.04 ± 0.04*	5.84 ± 0.04	0.19
Mineral composition (mg/100 g dry base)			
Calcium	322.84 ± 3.12*	66.12 ± 1.81	6.00
Copper	0.56 ± 0.07	0.64 ± 0.08	0.32
Iron	3.12 ± 0.10	3.01 ± 0.10	0.49
Magnesium	78.86 ± 0.61*	75.69 ± 0.51	1.71
Manganese	0.49 ± 0.00	0.72 ± 0.03*	0.08
Potassium	1593.07 ± 8.95	1850.58 ± 17.22*	60.52
Sodium	5.86 ± 0.53	6.38 ± 0.77	1.27
Zinc	5.90 ± 0.05*	4.77 ± 0.21	0.40

Results are presented as mean ± standard deviation. Proximate composition and carbohydrates as glucose are expressed as g/100 g dry basis, while mineral composition is expressed as mg/100 g dry basis. Initial moisture of samples: corm = 74.86 ± 0.24% and cormel = 72.97 ± 0.88%. Available carbohydrates were calculated by difference, subtracting protein, lipids, ash, and total dietary fiber from the initial dry weight of the sample. *LSD* least significant difference between samples by Student’s t-test; * indicates significant difference at *p* < 0.05.

CL exhibited a higher ash content (7.14 ± 0.13 g/100 g dry basis) than C (6.63 ± 0.09 g/100 g dry basis), with a significant difference between the fractions (*p* < 0.05). C and CL did not differ in lipid content (*p* > 0.05), and both showed values similar to those reported in the literature, which range from 0.77 to 1.26% [15]. The protein content of CL was 7.37 ± 0.11 g/100 g dry basis, whereas C contained 5.83 ± 0.21 g/100 g dry basis. These values are close to the range reported in previous studies on taro proximate composition, in which protein contents ranged from 4.03 to 6.22% [15]. The higher protein and ash contents observed in cormels may contribute to their nutritional value, particularly when used as flour or as an ingredient in plant-based food formulations.

In contrast, C showed a significantly higher total dietary fiber content than CL, with values of 23.27 ± 1.04 and 17.36 ± 0.50 g/100 g dry basis, respectively. Both values were higher than those reported for taro flours obtained by different processing methods, which ranged from 12.80 to 14.00% [16], and higher than the 8.24% reported by Pérez et al [17]. The higher dietary fiber content observed in corms may be associated with differences in the developmental stage and physiological function of these storage organs. While the corm represents the primary storage organ of the plant, cormels are lateral structures that exhibit distinct growth dynamics and reserve accumulation patterns [11]. Changes occurring during the development of taro storage organs are accompanied by important metabolic and compositional modifications [12], which may contribute to differences in dietary fiber content between corms and cormels. From a nutritional and technological perspective, the high fiber content of C is relevant because dietary fiber can contribute to water retention, texture formation, and modulation of starch accessibility during digestion.

The fiber and carbohydrate contents also suggest that both corms and cormels may be suitable raw materials for starch and mucilage recovery. Taro has previously been described as a potential source of mucilage, with reported contents ranging from 3.00 to 19.00% [6]. Mucilages, together with pectins and gums, are considered soluble non-digestible polysaccharides and may be included within the dietary fiber fraction, with potential effects on digestion and nutrient absorption [18]. Therefore, the compositional profile observed in both fractions supports their potential use in the development of ingredients with nutritional and techno-functional relevance.

Total carbohydrates did not differ significantly between C and CL (*p* > 0.05). However, C exhibited a significantly higher reducing sugar content (6.04 ± 0.04 g/100 g) than CL (5.84 ± 0.04 g/100 g) (*p* < 0.05). The higher reducing sugar content observed in C may be associated with differences in carbohydrate partitioning between soluble sugars and storage polysaccharides. During the development of taro storage organs, sucrose transported from source tissues is progressively converted into starch through coordinated metabolic pathways involved in storage organ filling [12, 19]. Therefore, the lower reducing sugar content observed in CL may indicate a greater conversion of soluble sugars into storage starch, whereas C retained a higher proportion of carbohydrates in the form of reducing sugars. Reducing sugars are mainly associated with simple sugars such as glucose and fructose, which may influence sweetness and technological behavior during thermal processing. In starch-rich matrices such as taro, these sugars may also promote browning reactions and aroma compound formation during heating [20].

The calcium content of C was particularly high, reaching 322.84 ± 3.12 mg/100 g dry basis, compared with 66.12 ± 1.81 mg/100 g dry basis in CL. This difference may be related to the limited phloem mobility of calcium, which is transported predominantly through the xylem and is not readily redistributed after deposition in plant tissues [21]. Previous studies on taro have shown that calcium distribution within storage organs is not uniform and may vary according to tissue type and developmental characteristics [3]. Therefore, the higher calcium content observed in C may reflect differences in mineral deposition and tissue organization between the primary storage organ and the lateral cormels.

Conversely, CL exhibited a higher potassium content (1850.58 ± 17.22 mg/100 g dry basis) than C (1593.07 ± 8.95 mg/100 g dry basis), with a significant difference between the fractions (*p* < 0.05). Considering the recommended potassium intake of 3500 mg/day for adults established by the World Health Organization [22], both fractions may contribute to dietary potassium intake, particularly when incorporated into food products as dry ingredients.

Overall, C and CL showed complementary nutritional characteristics. C was distinguished by its higher total dietary fiber, calcium, magnesium, and zinc contents, whereas CL was characterized by higher protein, ash, available carbohydrates, manganese, and potassium contents. These differences support the differentiated or combined use of both fractions as plant-based ingredients for food applications.

### Microstructural Characterization

**Fig. 2:**
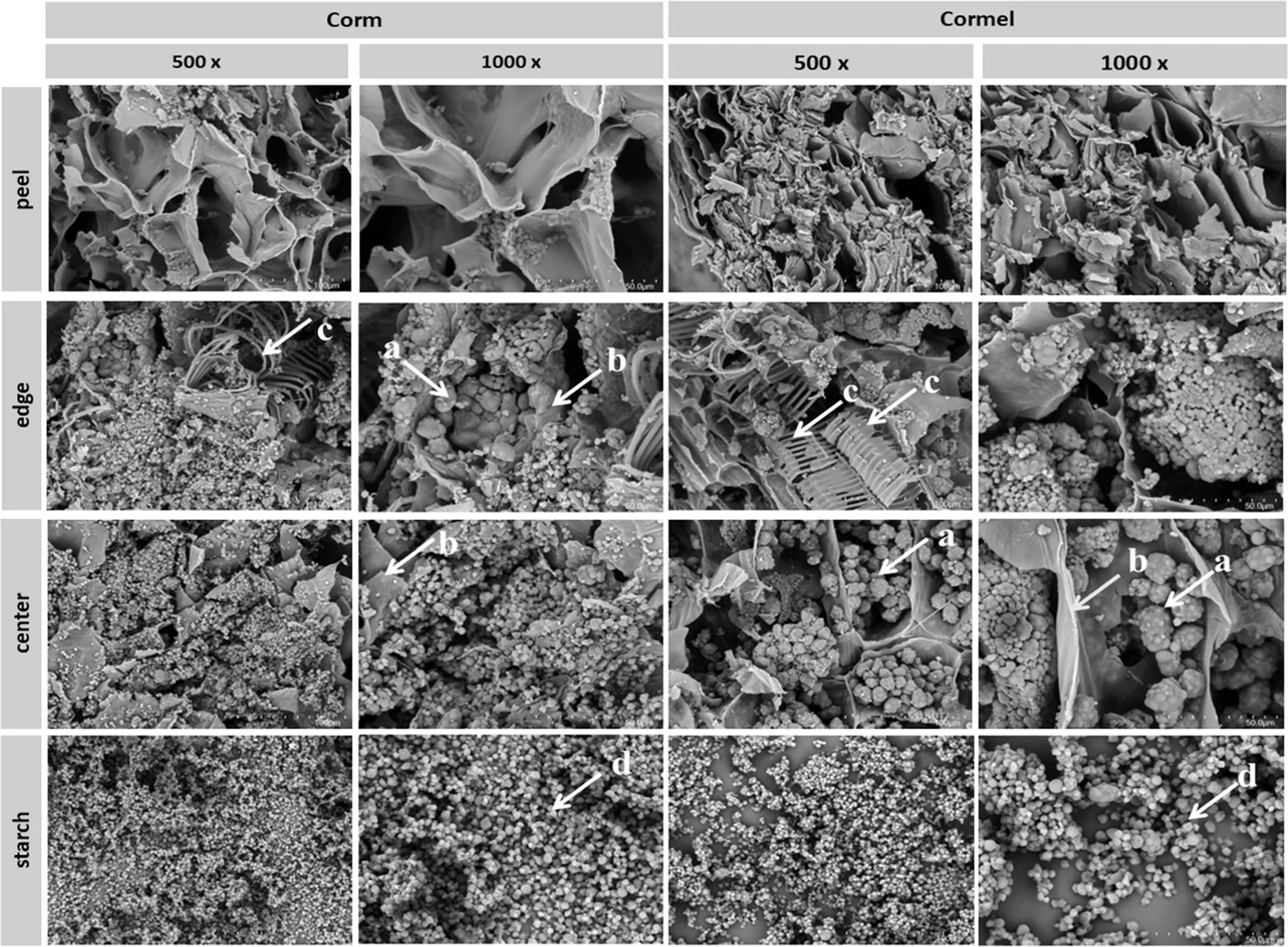
SEM images of freeze-dried sections and isolated starches from taro corms and cormels. Images show peel, edge, center, and isolated starch fractions at 500× and 1000× magnifications. Arrows indicate: (**a**) starch granule aggregates; (**b**) storage parenchyma cells surrounding starch granules; (**c**) elongated helicoidal structures compatible with xylem tracheary elements; and (**d**) isolated starch granules

Figure 2 shows SEM images of freeze-dried sections of C and CL of taro, including peel, edge, center, and isolated starch fractions. In both samples, starch granules were more abundant in the central region, whereas in the peel and edge regions they appeared embedded within a more complex fibrous matrix. This structural arrangement may hinder starch separation during extraction, but it may also influence starch accessibility in food matrices due to the physical association between starch granules and cell wall components.

Starch granules (Fig. 2a) were mainly organized in clusters or aggregates, with predominantly sub-spherical to polyhedral morphology, small size, rough surface, and slightly irregular contours. These characteristics are consistent with previous descriptions of taro starch granules as compact aggregates with irregular, cubic, or polygonal shapes and diameters commonly smaller than those of starches from arrowroot, cassava, Chinese yam, beans, corn, and potato [23]. In the present study, starch granules in C appeared more continuous and densely packed, with less defined aggregation and a greater presence of fibrous structures than those observed in CL.

The micrographs also revealed starch granules delimited by storage parenchyma cells (Fig. 2b), whose walls are composed mainly of structural polysaccharides and may contribute to the dietary fiber fraction. The morphology of starch granules is associated with their development within amyloplasts, where progressive growth and spatial compression can promote the transition from rounded to more polyhedral shapes [19]. In addition, elongated rigid structures with a helicoidal pattern were observed, mainly in the edge region near the center (Fig. 2c). These structures are compatible with xylem tracheary elements, characterized by thickened and lignified cell walls, as previously described for plant tissues [21, 24, 25]. Their preservation after freeze-drying indicates high structural resistance and supports the contribution of non-starch polysaccharides to the organization of the taro matrix.

The coexistence of small starch granules and fibrous structures is relevant from both technological and nutritional perspectives. Small starch granules have a high surface area, which may favor hydration, interaction with other food matrix components, and enzymatic susceptibility [26]. However, when starch granules remain physically embedded within cell wall material, enzyme access may be limited, potentially influencing starch digestion kinetics [27]. Regional differences within taro corm tissues have also been reported by Yu et al [28], who observed that starch granules from the middle region of taro corms had larger diameters and higher crystallinity than those from upper and basal regions, affecting texture and sensory quality.

The morphology of starch granules isolated from corms (CS) and cormels (CLS) is summarized in Table 3. CS exhibited significantly higher (*p* < 0.05) mean diameter (2.57 ± 0.98 μm), perimeter (10.12 ± 4.37 μm), and area (5.21 ± 3.98 μm²) than CLS (diameter = 2.02 ± 0.77 μm, perimeter = 8.07 ± 3.97 μm, and area = 3.25 ± 2.59 μm²). CS granules also showed greater dimensional variability, whereas CLS granules were more homogeneous. Circularity did not differ significantly between the samples, indicating that both starches consisted of irregular and non-perfectly spherical granules, as also observed in the scanning electron microscopy (SEM) images (Fig. 2d).

**Table 3:** Morphology of starch granules isolated from taro corms (CS) and cormels (CLS)

Morphology	CS	CLS	LSD
Average diameter (µm)	< 0.67–5.56 >	< 0.57–4.70 >	
	m = 2.57 ± 0.98*	m = 2.02 ± 0.77	0.16
Grain diameter distribution (µm)	Relative frequency (%)		
[0.50–1.00>	2.61	4.35	
[1.00–2.00>	26.09	49.13	
[2.00–3.00>	42.61	36.09	
[3.00–4.00>	18.26	7.83	
[4.00–5.00>	7.83	2.61	
[5.00–5.70>	2.61	0.00	
Perimeter (µm)	< 2.44–28.15 >	< 2.26–24.60 >	
	m = 10.12 ± 4.37*	m = 8.07 ± 3.97	0.76
Area (µm²)	< 0.30–19.17 >	< 0.20–14.89 >	
	m = 5.21 ± 3.98*	m = 3.25 ± 2.59	0.62
Circularity (µm²/µm²)	< 0.15–0.87 >	< 0.11–0.87 >	
	m = 0.60 ± 0.16	m = 0.61 ± 0.17	0.03

Results are presented as range < minimum–maximum > and mean ± standard deviation. Distribution data are presented as relative frequency (%). *LSD* least significant difference between CS and CLS by Student’s *t*-test; * indicates significant difference at *p* < 0.05. Circularity is dimensionless and ranges from 0 to 1, where 1 indicates a perfect circle.

The predominance of small starch granules in both CS and CLS suggests potential for food applications requiring smooth texture and homogeneous gels, such as creams, sauces, desserts, and other structured products. Similar ranges have been reported for taro starches from different origins. Tu et al. [29] described taro starch granules with diameters from 0.48 to 7.21 μm and predominantly polyhedral shapes with irregular surfaces, whereas another study on taro cultivated in Hunan, China, reported irregular polygonal granules with average diameters between 1.30 and 2.20 μm [30]. These values are comparable to those observed in the present study and reinforce the consistency of taro starch morphology across different varieties and cultivation regions. The small size of taro starch granules may also favor their use as thickeners, stabilizers, or texture-modifying agents due to their high surface area and interaction capacity within food matrices [31, 32].

### Pasting Properties and Gel Hardness

Table 4 presents the pasting properties and gel hardness of taro corms (C), cormels (CL), and their respective isolated starches, CS and CLS. The RVA curves obtained during the analysis are provided in Supplementary Figure S2.

**Table 4:** Pasting properties determined by RVA and gel hardness of taro corms (C), cormels (CL), corm starch (CS), and cormel starch (CLS)

Properties	C	CL	LSD	CS	CLS	LSD
Paste temperature (°C)	80.95 ± 0.48	80.77 ± 0.58	0.77	76.40 ± 0.43	79.30 ± 0.05*	0.70
Peak viscosity (cP)	1337.00 ± 12.17	2128.00 ± 10.82 *	26.10	1231.00 ± 27.51 *	1075.00 ± 11.00	47.50
Hold viscosity (cP)	1041.00 ± 16.52	1677.00 ± 10.54 *	31.41	717.00 ± 8.50	920.00 ± 3.00 *	14.46
Breakdown (cP)	296.00 ± 7.00	451.00 ± 13.53 *	24.42	513.33 ± 19.86*	155.00 ± 8.00	34.32
Final viscosity (cP)	1587.33 ± 12.22	2597.33 ± 41.86 *	69.90	1105.00 ± 10.82	1458.50 ± 12.50 *	26.50
Setback (cP)	546.33 ± 4.73	920.33 ± 31.34 *	50.81	387.33 ± 2.52	538.50 ± 9.50 *	15.75
Peak time (min)	4.71 ± 0.03	4.87 ± 0.00 *	0.06	4.40 ± 0.07	4.90 ± 0.03 *	0.12
Gel hardness (N)	2.83 ± 0.14	5.02 ± 0.20 *	0.40	3.67 ± 0.24	3.48 ± 0.08	0.41

Results are presented as mean ± standard deviation. *LSD* least significant difference between samples by Student’s *t*-test; * indicates significant difference at *p* < 0.05.

Among the whole fractions, CL exhibited higher peak viscosity (2128.00 ± 10.82 cP), hold viscosity (1677.00 ± 10.54 cP), final viscosity (2597.33 ± 41.86 cP), and setback (920.33 ± 31.34 cP) than C, with significant differences between the fractions (*p* < 0.05). The samples also differed in gel hardness after cooling, with CL showing higher values (5.02 ± 0.20 N) than C (2.83 ± 0.14 N). These results indicate that CL has a greater thickening capacity and forms more structured pastes and firmer gels, which may be advantageous for food products requiring consistency after heating and cooling, such as desserts, thick sauces, creams, and purées. However, CL also exhibited a higher breakdown (451.00 ± 13.53 cP) than C, indicating greater susceptibility to viscosity loss under heating and shear conditions. Therefore, although CL may provide higher viscosity and gel firmness, it may be less stable during intense thermal processing.

In contrast, C showed a lower breakdown value (296.00 ± 7.00 cP), suggesting greater resistance to granule disintegration during heating and shear. This behavior indicates better thermal and mechanical stability, which may be advantageous for products subjected to prolonged heating or intense agitation. The lower final viscosity (1587.33 ± 12.22 cP) and setback (546.33 ± 4.73 cP) observed for C also suggest a lower tendency for starch chain reassociation during cooling, which may help reduce excessive firming during storage.

Differences were also observed between CS and CLS. CS exhibited a lower pasting temperature (76.40 ± 0.43 °C) and higher peak viscosity (1231.00 ± 27.51 cP) and breakdown (513.33 ± 19.86 cP) than CLS, suggesting faster swelling but lower stability under heating and shear. Conversely, CLS showed higher hold viscosity (920.00 ± 3.00 cP), final viscosity (1458.50 ± 12.50 cP), setback (538.50 ± 9.50 cP), and peak time (4.90 ± 0.03 min), indicating greater resistance during heating and a stronger tendency to rebuild viscosity during cooling. This behavior may be related to differences in starch granule size and molecular organization following reassociation, as previously described for starch-based systems [33]. However, gel hardness did not differ significantly between CS and CLS, suggesting that the differences observed in RVA parameters did not translate into statistically distinct gel firmness under the tested conditions.

The comparison between the whole fractions and their respective isolated starches showed that CS and CLS generally exhibited lower peak viscosity, final viscosity, and setback values than C and CL, respectively. This behavior may be associated with the removal of non-starch components during starch isolation, including fibers, mucilage, proteins, and other polysaccharides, which can contribute to water retention and matrix structuring in whole flours. Interactions between starch and these components are known to influence the structural and rheological behavior of food systems, including viscosity development and paste stability [34].

The pasting temperatures of C and CL were close to 81.00 °C, whereas the isolated starches ranged from 76.40 °C for CS to 79.30 °C for CLS. These values are relatively high compared with those reported for some tuber starches, such as potato and cassava, which typically gelatinize at lower temperatures, generally between 55.00 and 70.00 °C [35]. In contrast, they are closer to the range reported for cereal starches, such as corn and rice, whose gelatinization or pasting temperatures may reach approximately 60.00–80.00 °C, depending on cultivar, starch structure, and analytical conditions [36]. This higher thermal requirement should be considered when selecting taro fractions or starches for food applications involving heating, thickening, or gel formation.

The morphological differences observed by SEM may have contributed to the distinct pasting profiles of CS and CLS. Although both starches exhibited predominantly polyhedral and aggregated granules, CS presented larger granules and greater dimensional variability than CLS. Previous studies have shown that larger starch granules tend to absorb water and swell more readily during heating, promoting higher peak viscosities but also greater susceptibility to disruption under thermal and shear stresses. In contrast, smaller and more homogeneous granules generally exhibit greater structural stability during heating and a higher ability to maintain or rebuild viscosity during cooling [30, 31]. Therefore, the structural differences observed are consistent with the distinct pasting behaviors exhibited by the two starches.

### Potential Food Applications

The nutritional, microstructural, and pasting properties observed in this study suggest distinct food applications for taro corms, cormels, and their isolated starches. Corms, characterized by higher dietary fiber, calcium, magnesium, and zinc contents, as well as lower breakdown and setback values, may be suitable for flour-based products requiring thermal stability and lower firming tendency during storage, such as porridges, broths, and ready-to-eat foods. In contrast, cormels, which showed higher protein, ash, available carbohydrate, manganese, and potassium contents, together with higher peak and final viscosities and greater gel hardness, may be more appropriate for products requiring structure and consistency after cooling, such as purées, creams, sauces, desserts, and gelled foods.

The isolated starches from corms and cormels, CS and CLS, also showed potential as food structuring agents due to their small granule size and distinct pasting behavior. Although starch isolation reduced viscosity compared with the whole fractions, these starches may be useful in food systems requiring smooth texture, moderate viscosity, and homogeneous dispersion. In addition, recovery of non-starch fractions, such as mucilage and dietary fiber, may further promote the integral use of taro and expand its application as a regional plant-based ingredient.

From a processing perspective, transforming taro corms and cormels into flours or starch-based ingredients may increase shelf life, reduce postharvest losses, and diversify their use beyond fresh consumption. Since taro is cultivated and consumed in several Latin American and Caribbean countries and territories, including Brazil, Colombia, Cuba, Haiti, Puerto Rico, and Venezuela, the valorization of corms and cormels may contribute to the diversification of regional plant-based foods and the broader use of underutilized crop fractions. This approach aligns with recent efforts to valorize regional crops in sustainable and gluten-free food applications, as reported for Andean pseudocereals [37].

## Conclusion

Taro corms and cormels cultivated in Brazil showed complementary nutritional, microstructural, and techno-functional properties, supporting their potential use as differentiated plant-based food ingredients. Corms were larger and more regular, with higher total dietary fiber, calcium, magnesium, and zinc contents, whereas cormels showed higher ash, protein, available carbohydrate, manganese, and potassium contents. The isolated starches, CS and CLS, presented small granules with sub-spherical to polyhedral morphology and rough surfaces, with CS showing larger granule dimensions than CLS.

The pasting behavior also indicated distinct technological applications for each fraction. Cormels showed higher viscosity development and gel hardness, suggesting suitability for products requiring structure and consistency, whereas corms presented lower breakdown and setback values, indicating greater stability during heating and a lower tendency toward retrogradation. Overall, the results provide a technical basis for the valorization of both taro fractions, particularly corms, which are less commercially valued, and support their use in the development of flours, starch-based ingredients, and food products with differentiated nutritional and technological properties. Future research should investigate the amylose content and thermal properties of taro starch using differential scanning calorimetry (DSC), as well as quantify oxalate levels in fresh corms and cormels. These complementary analyses could enhance the understanding of the relationships between starch composition, functionality, and processing behavior, while providing relevant information for the nutritional characterization and food applications of taro-derived ingredients. Furthermore, studies addressing the performance of these materials in specific food formulations, alongside assessments of digestibility, nutrient bioavailability, shelf life, consumer acceptance, and processing feasibility under local production conditions, would provide valuable insights for their broader application in food systems.

## Supplementary Information

Below is the link to the electronic supplementary material.

Supplementary Material 1 (DOCX 4.16 MB)

## Data Availability

All data supporting the findings of this study are available within the paper and its Supplementary Information.
