# Supplementary material for: Taro (Colocasia esculenta (L.) Schott) Corms and Cormels Cultivated in Brazil as Plant-Based Food Ingredients: Nutritional, Microstructural, and Pasting Properties
Source: Plant Foods Hum Nutr. 2026 Aug 18;81(3):110. doi: 10.1007/s11130-026-01544-7 (PMC13486127; doi:10.1007/s11130-026-01544-7)
Supplement: Supplementary file 1 [file 11130_2026_1544_MOESM1_ESM.docx]

**Supplementary Material**

**Detailed Material and Methodology**

**Plant Material**

*Taro* (Colocasia esculenta (L.) Schott) was obtained directly from a local producer in the Araújos neighborhood, rural area of the municipality of Bom Repouso, Minas Gerais, Brazil, at the geographic coordinates −22.482511, −46.150540. The plants were harvested nine months after planting and manually separated into corms (C) and cormels (CL). The samples were stored separately in fine jute bags under cool and dry conditions at room temperature and transported to the Laboratory of Cereals, Roots and Tubers II, Department of Food Science and Nutrition, School of Food Engineering, Universidade Estadual de Campinas (UNICAMP), Campinas, São Paulo, Brazil.

**Sample Processing**

Fresh tubers were washed under running water using a brush to remove external dirt, immersed in a 200 ppm sodium hypochlorite solution for 20 min, rinsed under running water, and stored at room temperature until analysis. Fresh C and CL samples were used for physicochemical characterization. Additional portions of each fraction were subjected to drying, freeze-drying, or starch extraction, according to the analytical requirements described below.

***Drying***

A total of 2 kg of each sample, C and CL, was dried. The tubers were peeled, chopped, and placed on stainless steel trays for drying in an oven at 65 °C until constant weight, according to method 925.09 [1]. The dried samples were ground, passed through a 60-mesh sieve, corresponding to 250 µm, and stored for nutritional composition (moisture, ash, lipids, protein, total dietary fiber, total carbohydrates, total and reducing sugars**)** and pasting properties.

***Freeze-Drying***

To preserve the native structure of the samples for microstructural characterization, two kilograms of each sample, C and CL, were sliced with the peel into 0.50 cm-thick pieces and immediately frozen at −20 °C in an ultra-freezer (Klimaquip, Model UK05B, Pouso Alegre, MG, Brazil). The slices were then freeze-dried using a Liotop LP820 freeze-dryer (Liobras, São Carlos, SP, Brazil). The freeze-drying process started at −30 °C for 18 h, followed by 0 °C for 18 h. The temperature was then gradually increased over 18 h until reaching 27 °C and maintained at 27 °C for 36 h, totaling 90 h of freeze-drying. The final pressure reached 88 µmHg. The samples were vacuum-stored at room temperature until microstructural analyses.

***Starch Extraction***

Starch extraction was performed according to Nascimento et al. [2], with modifications. Briefly, C and CL samples were separately peeled and cut into slices approximately 1 cm thick. The slices were homogenized in a blender (Hamilton Beach, Model FUR220CC5, Brusque, SC, Brazil) with distilled water at a sample-to-water ratio of 1:3 for 1 min at high speed. The resulting slurry was filtered through a 100 μm sieve to remove coarse fibrous material and transferred to a container containing distilled water at approximately ten times the volume of the filtrate to allow starch sedimentation. The filtrate was stored under refrigeration at 4 °C for 2 days. After sedimentation, the supernatant was discarded, and the sediment was repeatedly washed with distilled water and allowed to sediment until the supernatant became clear. The final sediment was dried in a forced-air oven at 50 °C until the moisture content was below 10%. Subsequently, the dried sediment was ground in a blender. The starch-rich fractions obtained from corms and cormels were designated as CS and CLS, respectively, and were used for microstructural and pasting property analyses. The extraction procedure involved physical separation steps, including homogenization, sieving, washing, and sedimentation, without additional chemical or enzymatic purification.

**Instrumental Color**

The color of the peel and pulp of fresh C and CL samples was determined using a Mini Scan HunterLab CR400 colorimeter (Konica Minolta Sensing Americas), calibrated with a white plate. Measurements were performed using the Hunter scale in the CIE L*a*b* color system. The L*, a*, and b* values were converted into RGB values using the Nix Sensor website and then converted into corresponding colors using Microsoft Excel, version 2408 (Microsoft Corporation, Redmond, WA, USA, 2024).

The external surface and pulp of C and CL were photographed under natural light using a 24.2 MP digital camera (Sony α6000 with 16–50 mm lens, f/3.5–5.6, at 50 mm), positioned 25 cm from the samples, with ISO 100, aperture f/6.3, and exposure time of 1/80 s. From the photographic images, the most representative image of each sample, external and internal, was selected to graphically represent rhizome color according to [3], with adaptations. Color calibration, white balance adjustment, and background removal were performed using Adobe Photoshop 2020.

Images were saved in PNG format and analyzed using ImageJ software, complemented by the Color Inspector 3D plugin, with processing defined by the Color Threshold function. Detailed RGB spectrum color information was obtained from Color Inspector 3D based on pixel distribution. RGB values were plotted in Microsoft Excel to visualize the most frequent colors on both the surface and pulp of the samples.

**Physicochemical Characterization**

Fresh samples of C and CL were individually evaluated for dimensioning, weight, specific gravity, moisture, dry matter, soluble solids, pH, and titratable acidity.

***Dimensioning***

From a total of 361 intact and healthy taro units, 19 units of each fraction, C and CL, were randomly selected, corresponding to the square root of the total number of samples, as indicated by [4]. The external surface and pulp of C and CL were photographed under natural light using a 24.2 MP digital camera (Sony α6000 with 16–50 mm lens, f/3.5–5.6, at 50 mm), positioned 25 cm from the samples, with ISO 100, aperture f/6.3, and exposure time of 1/80 s. The photographs were used to determine length, width, and regularity using ImageJ software (National Institutes of Health, Bethesda, MD, USA).

***Weight and Specific Gravity***

The samples were weighed, and specific gravity was determined based on the relationship between mass and volume of each sample, C and CL, with peel. The volume was measured by water displacement in a 1000 mL graduated cylinder, according to method 011/IV [5]. Specific gravity was expressed as g/mL. After peeling, part of each sample was used for moisture determination, and another part was used for soluble solids, pH, and titratable acidity analyses.

***Moisture and Dry Matter***

For moisture determination, 10 g of each sample, C and CL, were weighed and dried in an oven at 105 °C until constant weight, according to method 925.09 [1]. Dry matter was calculated as the difference between 100 and moisture percentage.

***Soluble Solids, pH, and Titratable Acidity***

A dilution of 20 g of each sample, C and CL, in 200 mL of water was prepared and blended using a Hamilton Beach blender (Hamilton Beach, Model FUR220CC5, Brusque, SC, Brazil). From this dilution, 10 mL were transferred to Falcon tubes and centrifuged at 3000 rpm for 10 min. The supernatant was used to determine soluble solids, expressed as °Brix, using a portable refractometer at room temperature, according to method 010/IV [5]. The same supernatant was used for pH measurement using a digital pH meter (HANNA Instruments, HI 2221, USA).

Titratable acidity was determined according to Method 6.1.2.1 for water-soluble acidity [5], based on titration of 10 mL of the initial diluted sample with 0.1 mol/L sodium hydroxide solution, using phenolphthalein as indicator until color change. The initial sample weight was corrected by multiplying by the dilution factors, and titratable acidity was then calculated.

**Nutritional Characterization**

The proximate composition of dried samples was determined by measuring moisture, according to method 925.09 [1]; ash, according to method 923.03 [1]; total lipids [6]; protein, according to method 2001.11 [1]; and total dietary fiber, according to method 985.29 [1]. Available carbohydrates were calculated by difference, using the following equation:

Available carbohydrates = 100 − moisture (%) − ash (%) − total lipids (%) − protein (%) − total dietary fiber (%).

Total carbohydrates as glucose were determined according to method 040/IV [5], with minor modifications. Three grams of sample were added to a beaker containing distilled water and 10 mL of 0.1 mol/L HCl. The solution was placed in a boiling water bath for 3 h. After cooling, the pH was adjusted to 7.0 using 0.1 mol/L NaOH. The sample was filtered, and distilled water was added. The solution was then titrated in an Erlenmeyer flask containing Fehling A and B solutions under constant heating and stirring. When the solution began to lose its blue color, two drops of 1% methylene blue were added, and titration continued until the blue color disappeared and a brick-red precipitate formed.

Reducing sugars as glucose were determined according to method 038/IV [5], with modifications. Six grams of sample were weighed and transferred to a 100 mL volumetric flask, and the volume was completed with distilled water. The sample was manually shaken for 3–4 min, filtered, and transferred to 50 mL Falcon tubes, which were centrifuged at 1300 rcf for 5 min. The supernatant was transferred to a burette and titrated in an Erlenmeyer flask containing Fehling A and B solutions under constant heating and stirring until the blue color disappeared and a brick-red precipitate formed.

The contents of calcium, copper, iron, magnesium, manganese, potassium, sodium, and zinc were determined by flame atomic absorption spectrometry (FAAS), using an AAnalyst 200 spectrometer. The mineralized samples were introduced into a nebulizer and mixed with an air–acetylene flame at approximately 2000 °C, with modifications according to [7].

**Microstructural Characterization**

***Scanning Electron Microscopy***

For scanning electron microscopy (SEM), freeze-dried slices of C and CL were used to evaluate the microstructural characteristics of different sample regions. Each slice was manually sectioned into three regions: peel, edge, and center. Fragments of approximately 1 cm were obtained from each region using a stainless-steel blade. In addition, the isolated starches obtained from C and CL, identified as CS and CLS, respectively, were also analyzed by SEM. All samples were mounted on circular aluminum stubs using double-sided carbon adhesive tape. The mounted samples were placed in a vacuum sputter-coating chamber until reaching approximately 1 × 10⁻¹ mbar. Subsequently, argon gas was introduced into the chamber, and the samples were coated with a thin gold layer for 2 min at approximately 18 mA using a sputter coater (EMITECH, model K450, Kent, UK). SEM images were obtained under high-vacuum conditions at an accelerating voltage of 15 kV, using magnifications of 500× and 1000×, using a TM 4000 Plus microscope (SU8000, HITACHI, Japan).

***Measurement of Starch Granules***

SEM images were analyzed using ImageJ software (National Institutes of Health, Bethesda, MD, USA). Images at magnifications of 1500×, 2000×, and 2500× were converted to 8-bit format, with contrast set to 192 and black-and-white adjustment. A total of 230 granules per sample were selected and measured. Starch granules were evaluated for mean diameter, calculated as the average of longitudinal and transverse diameters, perimeter, area, and circularity. Circularity values ranged from 0 to 1, where a value of 1.0 indicates a perfect circle.

**Pasting properties and gel hardness**

***Pasting Properties***

The pasting properties of C, CL, CS, and CLS were determined using a Rapid Visco Analyser, RVA-4500 model (Perten Instruments, Warriewood, Australia), using Thermocline for Windows software, version 3, according to method 76.21.01 [8]. Each sample was analyzed in triplicate.

A total of 3.50 g of sample, adjusted to 14% moisture, was weighed and added to 25 mL of water in an aluminum canister. Dispersion was performed using an Ultra-Turrax homogenizer, followed by a 10 min resting period for hydration. The aluminum canister was then coupled to the instrument’s acrylic paddle. The test was conducted using the Standard 1 program. The following parameters were recorded: pasting temperature (°C), peak time (min), peak viscosity (cP), hold viscosity (cP), breakdown (cP), setback (cP), and final viscosity (cP).

***Gel Hardness***

After RVA analysis, the cooked paste was kept in the same aluminum canister, covered with PVC film, allowed to cool, and stored under refrigeration at 5 °C for 16 h. After this period, the samples were maintained at room temperature for 1 h, and hardness was measured using a TA.XT2i texture analyzer (Stable MicroSystems, Surrey, England), according to [9]. A cylindrical acrylic probe with a diameter of 2.5 mm was used, with a test speed of 0.5 mm/s, a distance of 10 mm, and a 50 kg load cell.

**Statistical Analysis**

All analyses were performed in triplicate, and data were expressed as mean ± standard deviation. The results of physicochemical characterization, including instrumental color, specific gravity, moisture, dry matter, soluble solids, titratable acidity, and pH; nutritional characterization; microstructural characterization; and pasting properties were analyzed using Student’s *t*-test. The least significant difference (LSD) was calculated at a significance level of 5% (*p* < 0.05), using SISVAR statistical software, version 5.6, licensed by the Federal University of Lavras, Minas Gerais, Brazil. Graphs from pasting properties and relative frequency distributions of starch granules were generated using Microsoft Excel, version 2408 (Microsoft Corporation, Redmond, WA, USA, 2024).

**References**

1. AOAC (2011) Official methods of analysis, 18th edn. Association of Official Analytical Chemists, Washington, DC
2. Nascimento GC, Tagliapietra BL, Silva LR, Vieira FDP, Nolasco MVF, Nabeshima EH, Barros DR, Costa Pinto C, Michelon S, Clerici MTPS (2025) Technological and Functional Potentialities of Mairá (Casimirella rupestris) and Ariá (Goeppertia allouia) Starches. Plant Foods Hum Nutr 80:53. https://doi.org/10.1007/s11130-025-01293-z
3. Salvador-Reyes R, Furlan LC, Martínez-Villaluenga C, Dala-Paula BM, Nabeshima EH, Pinto CC, Souza SM, Pallone JAL, Clerici MTPS (2024) Peruvian fava beans for health and food innovation: physicochemical, morphological, nutritional, and techno-functional characterization. Food Res Int 192:114814. https://doi.org/10.1016/j.foodres.2024.114814
4. Cecchi HM (2003) Fundamentos teóricos e práticos em análise de alimentos, 2nd edn rev. Editora da Unicamp, Campinas. https://doi.org/10.7476/9788526814721
5. Instituto Adolfo Lutz (2008) Normas analíticas do Instituto Adolfo Lutz: Métodos químicos e físicos para alimentos, 4th edn. Instituto Adolfo Lutz, São Paulo
6. Bligh EG, Dyer WJ (1959) A rapid method of total lipid extraction and purification. Can J Biochem Physiol 37:911–917
7. Silva JGS, Orlando EA, Rebellato AP, Pallone JAL (2017) Optimization and Validation of a Simple Method for Mineral Potential Evaluation in Citrus Residue. Food Anal Methods 10:1899–1908. https://doi.org/10.1007/s12161-016-0748-3
8. AACC International (2010) Approved methods of analysis, 11th edn. AACC International, St. Paul, MN
9. Sandhu KS, Singh S (2007) Some properties of corn starches II: Physicochemical, gelatinization, retrogradation, pasting and gel textural properties. Food Chem 101:1499–1507. https://doi.org/10.1016/j.foodchem.2006.01.060

**Figures**

**Figure S1.** Morphological appearance of a harvested taro plant, showing the central corm or mother rhizome (a) and surrounding cormels or daughter rhizomes (b).

**Figure S2.** Pasting properties determined by RVA of *taro* corms (C), cormels (CL), corm starch (CS), and cormel starch (CLS)

.
